# Investigation of the Acceptance of Illness and Religious Coping Styles Among Newly Diagnosed Cancer Patients: Ankara, Turkey

**DOI:** 10.1007/s10943-025-02315-5

**Published:** 2025-04-24

**Authors:** Farhia Hassan, Nurhan Doğan

**Affiliations:** 1 Department of Medical Nursing, Amasya University Health Sciences Institute, Amasya, Turkey; 2 Department of Medical Nursing, Amasya University Faculty of Health Sciences, Amasya University İpekköy Campus Opposite Shell İpekköy/AMASYA, 05100 Amasya, Turkey

**Keywords:** Cancer, Acceptance of illness, Religious coping, New diagnosis, Nursing

## Abstract

This study was conducted to determine the acceptance of illness and religious coping styles of newly diagnosed cancer patients. The data were collected via descriptive characteristics such as the "Acceptance of Illness Scale" and "Brief Religious Coping Scale." In this study, individuals who were over 50 years of age, illiterate, not working, not smoking and not using alcohol had higher mean scores of positive religious coping, while those who were single and who received support from a psychologist in coping with illness had higher mean scores of negative religious coping (*p* < 0.05). Individuals who received support from a psychologist scored higher on the acceptance of illness scale than those who received support from themselves or their family (*p* < 0.05). There are significant relationships between acceptance of illness and positive religious coping (0.134) and negative religious coping (− 0.307) (*p* < 0.05). Age, marital status, employment status, smoking and alcohol use and receiving support from a person were found to be effective in religious coping, while receiving support from a psychologist was found to be effective in acceptance of illness. There is a relationship between positive and negative religious coping and acceptance of illness, and planning care accordingly is recommended.

## Introduction

Cancer remains one of the most common diseases in the world despite great advances in its diagnosis and treatment. At the beginning of the twentieth century, cancer ranked 7th and 8th among the leading causes of death worldwide, but today, it is second only to cardiovascular diseases in most parts of the world (Siegel et al., 2023). According to International Agency for Research on Cancer (IARC) 2020, approximately 19.3 million new cancer cases and approximately 10 million cancer-related deaths occur worldwide. According to forecasts, there will be approximately 28.4 million new cancer cases worldwide in 2040, an increase of 47% compared with 2020 (Tanır, 2023; WHO, 2024). Although these rates are increasing numerically, there are also challenging aspects of receiving a new cancer diagnosis for the individual. Patients experience anxiety and fear in the first stage of the diagnosis process, but in the following process, they experience the five stages of grief, also known as "Elisabeth Kübler-Ross's Method of Coping with Pain": "shock, denial, anger, depression and acceptance" (Kübler-Ross & Kessler, 2014). Accepting the diagnosis of cancer is reported to be important in psychological adaptation to the disease and in reducing cancer-specific distress (Secinti et al., 2019). Instead of accepting illness, cancer patients sometimes see their experience as their fate. Seeing the disease as fate is different from accepting the illness. Seeing the disease as a fate means believing that there is little or nothing that can be done to change or control the symptoms and quality of life (Aytaç & Kurtdaş, 2015). When individuals exhibit fatalistic behavior, they choose to remain helpless, hopeless and passive against the disease, and they give up and do not do anything to obtain satisfaction from life and stop trying anymore (İnal & Gürsu, 2023). Sometimes, a fatalistic approach can activate religious coping mechanisms in individuals (Karagöz, 2019).

### Religious coping

Religion is a sociological issue known for influencing people's decision-making and actions in many areas of social life. Inspiration and patience from religious sources are the first thing that can console a person in an unavoidable situation such as illness. Religion serves as a powerful source of inspiration in this regard (Karagöz, 2019; Koenig & Al Zaben, 2021; Koenig & Carey, 2024). When individuals face a life-threatening illness, they seek religious help, both physically and psychologically, and they are more attached to God/God to reduce the stress they experience, to accept the illness easily and to reduce depression (İnal & Gürsu, 2023; Koenig & Carey, 2024). In individual coping, the individual uses the skills and abilities developed in previous life (Güldü, 2023); in the case of religious coping, the person engages in rituals specific to his or her religion and relaxes himself or herself (Koç, 2020). In the literature, there are studies on cancer diagnosis and its aftermath, the relationship between the reactions of newly diagnosed cancer patients to cancer and their religious coping styles, and the effect of spiritual counseling services on accepting, understanding and explaining cancer disease (Altıntaş & Doğan, 2021; Gemalmaz & Avşar, 2015; İnal & Gürsu, 2023). However, no study has examined the acceptance of illness and religious coping styles of newly diagnosed cancer patients. This study was conducted to determine the acceptance of the illness and religious coping styles of newly diagnosed cancer patients.

## Method

### Study design

This study was conducted using a cross-sectional study design. Cross-sectional studies are a type of observational research that examines data from a population at a single point in time. These studies are commonly used to assess the prevalence of health conditions, explore factors that influence health, and characterize the population's traits. While they provide valuable preliminary insights for designing more advanced future research, establishing causal relationships is challenging. Additionally, cross-sectional studies are typically quick and cost-effective to carry out (Wang & Cheng, 2020). The cross-sectional design was chosen because it was compatible with the study's objective of assessing newly diagnosed cancer patients' acceptance of illness scale and religious coping scale styles at a specific point in time. This design allows data to be collected from a large sample in a limited period of time and to determine the current status of newly diagnosed cancer patients in terms of acceptance of illness and religious coping. By collecting data simultaneously across the target audience, the design allows for the effective identification of trends, correlations and areas requiring intervention.

This study followed the relevant EQUATOR guidelines and named the reporting method (Altman et al., 2008). The Strengthening the Reporting of Observational Studies (STROBE) checklist for cross-sectional studies was used to guide reporting (Vandenbrouckel et al., 2007).

### Population and sample

This cross-sectional study was conducted between April and May 2022 with 400 individuals over the age of 18, without communication problems, who were newly diagnosed with cancer and who applied to the oncology unit of a private hospital in Ankara. The data collection method used was convenience sampling in non-probability sampling methods. This technique involves selecting participants from easily accessible settings, such as hospitals, schools, databases, websites and membership lists, making them readily available for research (Stratton, 2021). Convenience sampling is simple and more time-efficient compared to other sampling methods (Stratton, 2021; Wang & Cheng, 2020). In this context, researchers reached out to and invited them to patients in the study. The adequacy of the sample size was determined using Cohen’s effect size (Cohen, 2013). G Power (3.1.9.7, Düsseldorf, Germany) program was used to calculate the sample size (Faul et al., 2007). The correlation between the levels of positive religious coping and acceptance of illness was effect size d = 0.36, with a 99% confidence interval (1–α) and 99% power (1–β), demonstrating that the sample size of 400 was sufficient.

### Data collection

The data in the study were collected by the researcher via a face-to-face interview technique. The data collection process took approximately 15–20 min. The study was conducted with individuals newly diagnosed with cancer who applied to the polyclinics of hospitals in Ankara.

- Newly diagnosed cancer patients,
- No communication problems,
- Verbal communication skills and individuals who agreed to participate in the study were included in the study.

Individuals who were previously diagnosed with cancer, recovered, experienced recurrence or developed secondary cancer were excluded from the study.

### Dependent and independent variables

Religious coping and acceptance of illness are the study’s dependent variables. The sociodemographic of individuals is independent variables.

### Data collection tools

In this study, data were collected via the "The Patient Information Form," which consists of 15 questions; the "Acceptance of Illness Scale (AIS)," which consists of 8 items; and the "Brief Religious Coping Scale (RCOPE)," which consists of 14 items.

### The patient information form

It was created by researchers by reviewing the literature (Altıntaş & Doğan, 2021; Gemalmaz & Avşar, 2015; İnal & Gürsu, 2023). It consists of a total of 13 questions, including questions concerning sociodemographic factors (age, marital status, education level), coping with illness and information about chronic disease.

### Acceptance of illness scale (AIS)

The Acceptance of Illness Scale (AIS) was developed by Felton, Revenson, & Hinrichsen (Felton et al., 2009). The Turkish validity and reliability of the scale were assessed by Besen and Esen in 2011 (Besen & Esen, 2011). The Acceptance of Illness Scale consists of eight items, each worth five points. The minimum score is 8, and the maximum score is 40. The Likert-type scale is proportioned according to 5-point agreement–disagreement. A low score indicates that illness is not accepted, and that poor adaptation is achieved. A high score indicates acceptance of the illness and the absence of negative feelings about the illness (Besen & Esen, 2011; Felton et al., 2009). The Cronbach’s alpha value of the scale is 0.83 (Felton et al., 2009). In this study, Cronbach’s alpha value of the scale was 0.85.

### Brief religious coping scale (RCOPE)

The scale was developed by Pargament et al. (Pargament et al., 1998). A validity and reliability study of the scale in Turkey was conducted by Ekşi (Ekşi, 2001). The scale consists of 14 items: 7 positive and 7 negatives. The four-point Likert-type scale consists of 14 items. It has two subdimensions: positive religious coping (items 1, 2, 6, 8, 9, 11 and 13) and negative religious coping (items 3, 4, 5, 7, 10, 12 and 14). It has two subdimensions: positive religious coping and negative religious coping. Positive religious coping involves cooperation, cooperation with God in problem solving, and suffering has a spiritual purpose. Negative religious coping is explained by a number of characteristics, such as spiritual detachment and a lack of belief in God's power and love or trying to find one’s own solutions without asking anything from God (Pargament et al., 1998). In the Turkish validity and reliability study of the scale, the Cronbach's alpha internal consistency value of the positive coping styles subscale was 0.64, and that of the negative coping styles subscale was 0.63 (Ekşi, 2001). In this study, Cronbach’s α reliability coefficient of the scale of the positive religious coping subdimension was 0.74, and that of the negative religious coping subdimension was 0.79.

In this study, precautions were taken to prevent data contamination by analyzing and calculating the subfactors individually, rather than computing an overall aggregate result, which could lead to biased findings. The 14-item Brief RCOPE used in the study, developed by Pargament et al. (Pargament et al., 1998), is an example of a proposed measure of religiosity/spirituality (Koenig & Carey, 2024).

### Statistical analysis

The SPSS 28.0 (Statistical Package for Social Sciences) program was used for data analysis. Descriptive statistics (number, percentage, mean, standard deviation) were used while evaluating the data. The reliability of the scale was examined with Cronbach's alpha. The Shapiro–Wilk test, skewness and kurtosis values and graphical examinations were used to test the normality of the quantitative data. The Mann–Whitney U test was employed to compare variables with a non-normal distribution in the paired analysis. The Kruskal–Wallis test was used to evaluate multiple groups of variables that did not follow a normal distribution. Dunn–Bonferroni test was used to determine between which two groups there were significant differences. The relationship between continuous variables was analyzed by Spearman correlation analysis. The statistical significance level was accepted as *p* < 0.05. The analysis of the study was carried out by independent statistics experts. There are no missing data in the study.

#### Ethical considerations

Before starting the research, ethics committee approval (approval date and number 01.04.2022 -3/40) was obtained from a university's non-interventional clinical research ethics committee, and written permission was obtained from the health directorate of the province where the research would be conducted and from the private hospital where the research would be conducted. Participants who volunteered to participate in the study were informed about the research, and the scale questions were applied after written informed consent was obtained. The research was conducted in accordance with the principles of the Declaration of Helsinki.

## Results

The mean age of the individuals who participated in the study was 52.8 ± 14.4 years. A total of 80.5% of the individuals were married, 45.8% had primary education, 63.0% did not smoke or drink alcohol, 89.5% learned the diagnosis from their first doctor, 92.5% received treatment, 73.3% did not work, and 89.7% received support to cope with the disease (Table 1).

**Table 1:** Descriptive characteristics of individuals (*n* = 400)

Characteristics	Min–Max	Median	Mean ± SD
Age	19.0–88.0	54.0	52.8 ± 14.4
BMI	16.5–47.8	24.8	25.2 ± 4.4
Duration of illness (days)	1.0–30.0	16.0	16.6 ± 8.7
Treatment duration (days)	1.0–18.0	7.0	7.4 ± 4.0
Acceptance of Illness Scale	8.0–40.0	20.0	21.0 ± 6.8
Positive Religious Coping	7.0–28.0	20.0	18.9 ± 5.5
Negative Religious Coping	7.0–20.0	7.0	8.4 ± 2.3

Characteristics		Frequency (F)	Percentage (%)
Age	≤ 50	169	42.3%
	> 50	231	57.8%
BMI	≤ 25	205	51.3%
	> 25	195	48.8%
Marital status	Single	31	7.8%
	Married	322	80.5%
	Widow	34	8.5%
	Divorced	13	3.3%
Education status	Illiterate	19	4.8%
	Primary education	183	45.8%
	High School	127	31.8%
	Higher Education	71	17.8%
Smoking and alcohol	Use	148	37.0%
	No	252	63.0%
Employment status	Employed	107	26.8%
	Not working	293	73.3%
The first person to make a diagnosis	Doctor	358	89.5%
	Family	42	10.5%
Receiving treatment	Yes	370	92.5%
	No	30	7.5%
Support in coping with the disease	Yes	359	89.7%
	No	41	10.3%
People who support you in coping with the illness	Psychologist	35	8.8%
	Family	318	79.5%
	Himself/herself	47	11.8%

SD: standard deviation, BMI: body mass index

Individuals over 50 years of age have higher mean scores of positive religious coping than individuals aged 50 years and younger (*p* < 0.05). The mean scores of the negative religious coping subdimension of individuals whose marital status was single were significantly higher. The Dunn–Bonferroni correction revealed that the significant difference was between the married and single groups (*p* < 0.05). Illiterate individuals have higher mean scores in positive religious coping than individuals with other educational levels. The Dunn–Bonferroni correction revealed that the significant difference was between the illiterate and higher education (*p* < 0.05). The positive religious coping scores were significantly higher for individuals who did not smoke or drink alcohol (*p* < 0.05). The positive religious coping scores of not working individuals are significantly higher (*p* < 0.05). The negative religious coping was higher in those who received support from a psychologist. The Dunn–Bonferroni correction revealed that the significant difference was between the psychologist and family (*p* < 0.05). There was no significant difference in positive and negative religious coping scale according to whom the individuals learned their diagnosis, whether they were currently receiving treatment and whether they received support in coping with the disease (*p* > 0.05, Table 2).

**Table 2:** Mean scores of the brief religious coping scale according to the descriptive characteristics of individuals (*n* = 400)

Scales variables	Characteristics	Frequency (F)	Min–Max	Median	Mean ± SD	Mean ranks	Test	p	Adj. significant difference	Adj. significant effect size
Age										
Positive Religious Coping	≤ 50	169	7.0–27.0	19.0	18.1 ± 5.7	184.57	− 2.369	**0.018**	–	–
	> 50	231	7.0–28.0	21.0	19.4 ± 5.3	212.15				
Negative Religious Coping	≤ 50	169	7.0–19.0	7.0	8.5 ± 2.3	209.97	− 1.551	0.121	–	–
	> 50	231	7.0–20.0	7.0	8.3 ± 2.3	193.57				
BMI										
Positive Religious Coping	≤ 25	205	7.0–27.0	20.0	18.9 ± 5.2	197.03	− 0.618	0.536	–	–
	> 25	195	7.0–28.0	21.0	18.9 ± 5.8	204.15				
Negative Religious Coping	≤ 25	205	7.0–19.0	7.0	8.2 ± 2.1	193.97	− 1.282	0.200	–	–
	> 25	195	7.0–20.0	7.0	8.6 ± 2.5	207.36				
Marital Status										
Positive Religious Coping	Single	31	8.0–27.0	17.0	17.5 ± 5.5	170.76	7.953	**0.047**	–	**–**
	Married	322	7.0–28.0	20.0	18.9 ± 5.5	200.66				
	Widow	34	7.0–25.0	22.0	20.7 ± 5.1	241.41				
	Divorced	13	7.0–25.0	17.0	16.8 ± 6.3	160.42				
Negative Religious Coping	Single	31	7.0–17.0	9.0	9.7 ± 3.1	256.84	10.731	**0.013**	Married- Single	**0.008 Effect size = 0.160**
	Married	322	7.0–20.0	7.0	8.2 ± 2.1	193.74				
	Widow	34	7.0–14.0	7.0	8.6 ± 2.4	210.16				
	Divorced	13	7.0–18.0	7.0	9.2 ± 3.5	208.42				
Education Status										
Positive Religious Coping	Illiterate	19	7.0–25.0	24.0	21.2 ± 5.4	256.76	10.745	**0.013**	Higher Education- Illiterate	**0.022 Effect size = 0.598**
	Primary education	183	7.0–26.0	21.0	19.3 ± 5.4	209.69				
	High School	127	7.0–27.0	20.0	18.7 ± 5.4	195.54				
	Higher Education	71	7.0–28.0	18.0	17.4 ± 5.7	170.61				
Negative Religious Coping	Illiterate	19	7.0–17.0	7.0	8.7 ± 2.9	206.08	4.953	0.175	–	–
	Primary education	183	7.0–20.0	7.0	8.2 ± 2.1	195.12				
	High School	127	7.0–19.0	7.0	8.3 ± 2.3	193.75				
	Higher Education	71	7.0–17.0	8.0	8.9 ± 2.5	224.94				
Smoking and Alcohol										
Positive Religious Coping	Yes	148	7.0–28.0	19.0	18.0 ± 5.9	184.02	− 2.196	**0.028**	–	**–**
	No	252	7.0–27.0	21.0	19.4 ± 5.2	210.18				
Negative Religious Coping	Yes	148	7.0–20.0	7.0	8.4 ± 2.3	194.19	− 0.105	0.387	–	**–**
	No	252	7.0–19.0	7.0	8.4 ± 2.3	204.21				
Employment Status										
Positive Religious Coping	Employed	107	7.0–27.0	18.0	17.4 ± 5.9	173.12	− 2.877	**0.004**	–	–
	Not working	293	7.0–28.0	21.0	19.4 ± 5.3	210.50				
Negative Religious Coping	Employed	107	7.0–17.0	7.0	8.3 ± 2.2	179.74	− 0.521	0.603	–	–
	Not working	293	7.0–20.0	7.0	8.4 ± 2.3	208.08				
The first person to make a diagnosis										
Positive Religious Coping	Doctor	358	7.0–28.0	20.0	18.7 ± 5.5	197.33	− 1.607	0.108	–	–
	Family	42	9.0–27.0	22.0	19.9 ± 5.5	227.49				
Negative Religious Coping	Doctor	358	7.0–20.0	7.0	8.3 ± 2.2	199.40	− 0.498	0.618	–	–
	Family	42	7.0–17.0	7.0	8.7 ± 2.6	209.90				
Receiving treatment										
Positive Religious Coping	Yes	370	7.0–28.0	20.0	18.8 ± 5.5	198.25	− 1.371	0.170	–	–
	No	30	9.0–25.0	21.5	20.2 ± 5.1	228.20				
Negative Religious Coping	Yes	370	7.0–20.0	7.0	8.4 ± 2.3	203.29	− 1.878	0.060	–	–
	No	30	7.0–14.0	7.0	7.7 ± 1.5	166.05				
Support in coping with the disease										
Positive Religious Coping	Yes	359	7.0–28.0	20.0	18.9 ± 5.4	199.33	− 0.603	0.546	–	–
	No	41	7.0–25.0	21.0	18.9 ± 6.3	215.38				
Negative Religious Coping	Yes	359	7.0–20.0	7.0	8.3 ± 2.2	199.58	− 0.518	0.604	–	–
	No	41	7.0–17.0	7.0	8.7 ± 2.6	208.51				
People who support you in coping with the illness										
Positive Religious Coping	Psychologist	35	7.0–26.0	20.0	17.3 ± 7.1	184.16	0.782	0.676	–	–
	Family	318	7.0–28.0	20.0	19.1 ± 5.2	200.70				
	Himself/herself	47	7.0–25.0	20.0	18.5 ± 6.2	223.15				
Negative Religious Coping	Psychologist	35	7.0–20.0	9.0	9.9 ± 3.5	251.49	9.350	**0.009**	Family- Psychologist	**0.007 Effect size = 0.156**
	Family	318	7.0–19.0	7.0	8.2 ± 2.1	194.64				
	Himself/herself	47	7.0–17.0	7.0	8.5 ± 2.5	202.19				

Bold values indicate significance at *p* = 0.05

^m^Mann–Whitney U test/^K^ Kruskal–Wallis test/post hoc: Dunn's multiple comparison test

Those who received support from a psychologist had higher scores on the acceptance of illness scale compared to those who received support from self and family. The Dunn–Bonferroni correction revealed that the significant difference was between family and psychologist support (*p* < 0.05).

There was a significant positive correlation between acceptance of illness and positive religious coping (0.134) and a significant negative correlation between negative religious coping and acceptance of illness (− 0.307) (*p* < 0.05, Table 3).

**Table 3:** Relationships between individuals' brief religious coping and acceptance of illness scale scores (*n* = 400)

Scales		1	2	3
1. Acceptance of Illness	r	1		
2. Positive religious coping	r	0.134^**^	1	
3. Negative religious coping	r	− 0.307^**^	− 0.569^**^	1

Spearman correlation, r: Spearman rank correlation coefficient; **p < 0.01; *p < 0 .05

## Discussion

When individuals are first diagnosed with cancer, they experience complex emotions such as confusion, anger, anxiety, fear, worry and grief. This is a difficult process, ranging from sadness and regret at the deterioration of one's health to seeing the process as a struggle or dismissing the significance of the disease (Karakartal, 2018; Kübler-Ross & Kessler, 2014). Acceptance of the disease is important for cancer patients, as it is for all individuals diagnosed with chronic diseases, because acceptance of the illness affects the patient's attitude and subsequent coping strategy (Erbay et al., 2017; Karakartal, 2018; Lotfi & Karataş, 2020). Acceptance of the illness affects various aspects of the patient's life, such as physical, mental, emotional, social and spiritual well-being (Erbay et al., 2017; Lotfi & Karataş, 2020). Çevik et al. reported that factors such as the sociodemographic characteristics of individuals (income, age, gender, education level, etc.) affect the process of accepting the illness (Çevik et al., 2022). In this study, it was determined that the sociodemographic characteristics (such as age, sex and education level) of newly diagnosed individuals were not effective in accepting the illness. The reason for the different results reported in the literature is that the individuals were newly diagnosed.

In Altıntaş's study on the reactions of newly diagnosed breast cancer patients to the disease and their religious coping, 98% of the patients learned the first diagnosis from the physician, 61.3% felt helpless and cried when they first learned about the disease, and 26.7% thought it was fate and was patient (Altıntaş & Doğan, 2021). In the present study, 89.5% of individuals learned the diagnosis from the first doctor, and when they heard the first diagnosis, they were shocked and did not want to believe it; then, they believed that it was their fate. The acceptance of illness scale score of 21.0 ± 6.8, which is close to the middle level, also supports this result.

A study conducted by Gemalmaz and Avşar with newly diagnosed cancer patients reported that individuals preferred to share their diagnosis with friends rather than with family members and that family members concealed the diagnosis from the patient if the individual's physician told the diagnosis of cancer to a family member (Gemalmaz & Avşar, 2015). As a result, 10.5% of the individuals learned their diagnosis from their family members. They stated that when the diagnosis was first told to the family, it was hidden from the patients, but they themselves felt it. When a life-threatening disease such as cancer is diagnosed, the individual is faced with a distressing situation, and it may not make sense to him/her from whom he/she first learned the diagnosis.

In the Gemalmaz and Avşar study, the first reaction when the diagnosis of cancer was first learned was a fatalistic approach (Gemalmaz & Avşar, 2015). In a study conducted by Gemalmaz and Avşar with newly diagnosed cancer patients, the interest of their families increased after the diagnosis of cancer, and they were satisfied with this development. However, it was also reported that they expressed that they were uncomfortable with the excessive interest exhibited by their families (Gemalmaz & Avşar, 2015). Altıntaş and Doğan (2021) reported that 97.3% of individuals diagnosed with cancer received support for coping and that 72.0% of them received this support from their families, and were satisfied with this situation and facilitated their adaptation to treatment (Altıntaş & Doğan, 2021). In the present study, 89.7% of the patients received support, and 79.5% received family support. Those who received support accepted their illness more than those who did not and did not have negative feelings about the illness. Those who received support from psychologists accepted their illness more than those who received support from family and did not have negative feelings about the illness. The reason for this may be that although the family has an important place in the life of the individual, they may have difficulty accepting and coping with the disease when they are not professionally supported.

It is important to help patients adapt to their daily lives and disease and to understand the emotional reactions of individuals. For individuals to cope positively with this process, meeting their emotional needs and providing environmental support is important. Even if the support given to the patient does not eliminate the stressor, it facilitates emotional adaptation to the disease, has a positive effect on coping with the disease and provides morale to the individual (Ateş, 2019; Dogan & Tan, 2019; Molina-Mula & Gallo-Estrada, 2020). However, family members feel that they are too hard on the patient, and patients feel that they are a burden to their families, who are grieving and making efforts for them.

When dealing with a personally threatening situation, such as a cancer diagnosis, patients often use religious coping (Coşgun & Tokur, 2022). In this study, it was determined that individuals used the religious coping method positively. Individuals over 50 years of age, widowed, illiterate, non-smokers, non-alcohol users and non-workers used positive religious coping methods. Another study reported that individuals adopted both positive and negative religious coping styles, showed good levels of spirituality, and positively affected their self-management (Karatana & Yıldız, 2024). Sabancıoğulları and Yılmaz reported that patients' level of hope, age and general health status significantly affected their positive religious coping methods. In the same study, it was found that sociodemographic and disease characteristics did not affect the religious coping levels of patients and that individuals living with their spouses and children used religion positively (Sabanciogullari & Yilmaz, 2021).

In Altıntaş's study, the relationship between religious coping and age groups was examined, and it was found that the positive religious coping score of those aged 60 years and over was higher than that of the other age groups, and while the positive religious coping score increased with age, the negative religious coping score decreased (Altıntaş & Doğan, 2021). In Ayten and Yıldız's study, positive religious coping attitudes were greater at older ages than at younger ages (Ayten & Yıldız, 2016). In this study, individuals over the age of 50 years were found to use the religious coping method positively.

In this study, a positive relationship was found between accepting illness and positive religious coping, and a negative relationship was found between negative religious coping. Another study reported that there was a negative relationship between patients' level of acceptance of the illness and negative religious coping (Şentürk et al., 2022). These results suggest that negative or positive religious coping styles may affect acceptance of the illness. The findings of the study support the literature. Individuals experiencing cancer have positive effects on discovering the meaning of life, adapting better to treatment, looking at the future with more hope and improving their quality of life. On the other hand, religious coping strategies are the most commonly used method among patients who believe in God and tend to increase their psychological–spiritual well-being (de Medeiros et al., 2024). There is evidence that the spiritual needs of patients diagnosed with cancer are not adequately addressed by health professionals (Otuzoğlu, 2020). Therefore, it is important for health professionals to understand the factors affecting the process of accepting illness and religious coping in newly diagnosed patients and to provide psychosocial and spiritual support to individuals.

## Limitations and future research

The results are limited to the patient population in this study and cannot be generalized to all newly diagnosed cancer patients. In addition, it is a cross-sectional descriptive study, so causal relationships between variables cannot be established. Since the data were collected through face-to-face interviews, individuals may not have been able to clearly express their views on religious coping. The relationship between religion/art and health outcomes is challenging to assess quantitatively without influencing mental health indicators (Koenig & Carey, 2024, 2025). One approach could be asking individuals to categorize themselves into one of four groups: (i) spiritual and religious, (ii) spiritual but not religious, (iii) religious but not spiritual and (iv) neither. While this avoids overlap with mental health, it still depends on personal definitions of spirituality and religion, making it difficult to quantify. Future research should explore these issues using qualitative or mixed methods (Koenig & Carey, 2024).

## Conclusion

In newly diagnosed cancer patients, religious coping was found to be affected by demographic factors such as smoking and alcohol use, education, marital status, employment status and the person providing psychological support. Therefore, it is important for nurses to evaluate the religious coping methods of individuals, encourage positive religious coping strategies and support them in managing negative religious coping methods. Positive religious coping strategies were more common in people who did not smoke or drink alcohol. Individuals who do not use tobacco or alcohol may be more inclined to turn to social and religious support systems. Religious coping can provide spiritual and community support, which can enhance positive religious coping skills. Therefore, individualized support programs can be provided to facilitate coping according to individuals' religious approaches, and it will be important to cooperate with professionals who will provide psychological support in this regard.

It was determined that receiving support from a psychologist was effective in accepting the disease. In this study, where professional support was important, increasing awareness and providing training to healthcare professionals about both religious coping strategies and acceptance of the disease would be useful. This will help health professionals communicate more effectively with patients, provide appropriate support, and facilitate access to support by patients. There is a relationship between acceptance of illness and positive and negative religious coping. For newly diagnosed patients, establishing religious or spiritual support groups that can strengthen social support networks, improve coping processes, and provide patients with access to community resources is important.

## Data Availability

The data generated and/or analyzed during the current study are not publicly available, but data may be provided by the corresponding author upon reasonable request.
